# Negative life events and aggression among Chinese rural left-behind adolescents: do self-esteem and resilience mediate the relationship?

**DOI:** 10.1186/s12888-023-04587-1

**Published:** 2023-03-15

**Authors:** Si Lan Yang, Chu Xia Tan, Juan Li, Jie Zhang, Yi Ping Chen, Yi Fei Li, Ying Xiang Tao, Bi Yun Ye, Shi Hao Chen, Hui Yuan Li, Jing Ping Zhang

**Affiliations:** 1 The First Hospital of Jiaxing, Affiliated Hospital of Jiaxing University, Zhejiang, China; 2 Xiangya Nursing School of Central South University, 172 Tong Zi Po Road, Changsha, 410000 Hunan China; 3 The Nethersole School of Nursing, Faculty of Medicine, The Chinese University of Hong Kong, Shatin, N.T., Hong Kong SAR, China

**Keywords:** Rural left-behind adolescents, Resilience, Self-esteem, Negative life events, Aggression, Mediation

## Abstract

**Background:**

Left-behind adolescents (LBAs) are adolescents aged 11–18 years who are separated from their parents and left behind in local cities by one or both parents for a period of more than 6 months. LBAs in rural areas are likely to engage in aggressive behavior, which can affect interpersonal relationships, reduce academic performance, and even lead to anxiety and depression. To our knowledge, no studies have examined the mediating effect of resilience and self-esteem on the relationship between negative life events and aggression among Chinese rural LBAs. Therefore, this study aimed to explore the relationship between negative life events and aggression among Chinese rural LBAs and how self-esteem and resilience mediate the association.

**Methods:**

Using a stratified random sampling method, 1344 LBAs in Hunan Province of China were investigated. Information was collected by a self-designed sociodemographic questionnaire, Adolescent Self-Rating Life Events Checklist, Resilience Scale Chinese Adolescent, Rosenberg Self-Esteem Scale and Aggression Scales to assess the psychology of LBAs. Data analysis was conducted using descriptive statistics, Pearson correlation, and regression analysis to estimate direct and indirect effects using bootstrap analysis.

**Results:**

Negative life events were significantly related to self-esteem (*r* = − 0.338), resilience (*r* = − 0.359), and aggression (*r* = 0.441). Aggression was directly affected by self-esteem (*β* = − 0.44) and resilience (*β* = − 0.34). Negative life events were not only directly related to aggression (*β* = 0.34, 95% CI: 0.275 ~ 0.398) but also showed an indirect effect on aggression through self-esteem and resilience. The direct effect, total effect and indirect effect of negative life events on aggression through self-esteem and resilience were 0.3364, 0.4344 and 0.0980, respectively. The mediating effect of self-esteem and resilience accounted for 22.56% of the relationship between negative life events and aggression.

**Conclusions:**

We found that self-esteem and resilience mediated most negative life events on aggression. It is imperative for educators and families to improve LBAs’ self-esteem and resilience to reduce the occurrence of aggression. Future intervention studies should be designed to strengthen self-esteem and resilience.

## Background

With rapidly increasing economic development and the current imbalanced regional economic situation in China, a great number of migrant workers from rural areas migrate to major cities for better job opportunities and pay, so children must be separated from their parents [1]. Children who are left behind in undeveloped areas by one or both parents for over 6 months are known as left-behind children [2]. Left-behind adolescents (LBAs) account for 29.62% of the total number of left-behind children [3] and are defined as children aged 11–18 years who are separated from their parents and left behind in local cities by one or both parents for more than 6 months and who have not lived with their parents for over 6 months [4]. Adolescence is an essential period of socialization in a youth’s life, and LBAs are likely to have psychological and interpersonal problems, such as self-hatred [1] and low friendship quality, and to encounter other negative events because of their parents’ absence [5].

Events or situations that challenge, threaten, damage, or exceed the physical and mental capacity of individuals are called negative life events [6]. LBAs may experience more negative events because they lack parental supervision and care [7]. For example, they have worse nutritional status, do more housework [8] and have no choice but to care for aged or sick caregivers [9]. Beyond that, they feel a higher degree of pressure due to negative life events (e.g., interpersonal relationships, study pressure, punishment and sense of loss) [10]. In China, LBAs are easily influenced by a series of negative life events and become aggressive. Aggression refers to destructive behaviors against oneself or others, including physical and verbal aggression [11], and are an important predictive factor in many problem behaviors in adolescents [12]. LBAs in rural areas are more likely to engage in aggressive behavior than average students because of the long-term separation from parental supervision and family care [13], with an incidence of 35.2% for aggressive behavior of LBAs [14]. Aggression can cause negative emotions such as anxiety and depression in LBAs [15] and can even lead to serious consequences, such as personality distortions, behavioral disorders, and a higher risk of suicide [16], seriously affecting their healthy development and social stability [17]. The “general aggression model” suggests that stressful life events are primary circumstantial factors significantly associated with aggression [18]. Similarly, a previous study showed that an ill sibling, failing a grade, and a lack of popularity with peers are all positively associated with subsequent bullying behaviors [18]. The occurrence of negative life events is difficult to avoid. Therefore, it is important to clarify the influencing factors of aggression caused by negative life events and implement corresponding interventions.

Researchers have found that some children develop well in stressful situations, even beyond the level of normal children, because of resilience [5]. Resilience is reflected in a tenacious attitude and is the ability to adapt well and maintain or restore mental health in the face of pressure or adversity [19]. Previous studies have demonstrated that resilience can attenuate the mental health problems of young adults who experienced childhood adversity (e.g., abuse, neglect, and household dysfunction) [20]. There is a significant negative correlation between negative life events and resilience in adolescents, and aggression was significantly associated with adolescents’ resilience in one study [21]. In summary, resilience may play a mediating role between negative life events and aggression.

Self-esteem is a state of admiration and acceptance due to the approval of the concept of self that is achieved as a result of the self-evaluation of the individual [22]. Masten noted that children’s positive self-evaluation, such as self-confidence, high self-esteem and self-efficacy, is considered an important individual feature that plays a positive predictive role in the development of resilience. Higher self-esteem means more confidence in one’s ability and more positive strategies, which are conducive to the development of resilience [23]. Self-esteem is a variable that may potentially influence behavioral development and is particularly important for adolescents, who experience a process of identity development [24]. As an internal protective factor of resilience, it may not only improve individuals’ level of mental toughness but also enable them to cope with pressure and setbacks in life [25]. Other studies have shown that self-esteem, an important concept of individual self-concept, may be an important predictor of resilience, and self-esteem can positively predict adolescents’ resilience [26]. In addition, prior research has shown that self-esteem is a crucial individual variable that is closely related to life events [27]. The theory of antisocial personality disorder suggests that a threat of self-esteem is disturbing to people who are prone to violence and often causes a strong sense of shame [28]. When the adjustment of psychological resilience fails, individuals may rely on aggressive behavior to try to restore their sense of self-worth due to the loss of self-esteem. Therefore, resilience might have a potential mediating role between self-esteem and aggression. Many studies have also found that the relationship between self-esteem and aggression may be regulated by psychological mechanisms [29]. For example, one researcher reported that prisoners’ emotional regulation plays a mediating role between self-esteem and aggressive behavior [30]. Another study reported the mediating role of depression and family support between self-esteem and aggressive behavior in adolescent groups [31]. In summary, self-esteem and resilience play mediating roles. There is also a sequential mediating role: negative life events may affect aggression through the sequential mediating role of self-esteem and resilience. However, to the best of our knowledge, there is little research on the mediating role of resilience and self-esteem in the relationship between negative life events and aggression among Chinese rural LBAs.

The separation of parents and children is widespread in the Chinese social environment. It is impossible to eliminate the occurrence of negative life events in LBAs, but it can be changed with appropriate interventions for resilience and self-esteem [32]. We aimed to explore the association between negative life events and aggression among Chinese rural LBAs and how resilience and self-esteem mediate the association based on a structural equation model and to provide a conceptual reference framework for prevention and intervention to help LBAs living in rural areas cope with aggression.

**Fig. 1:**
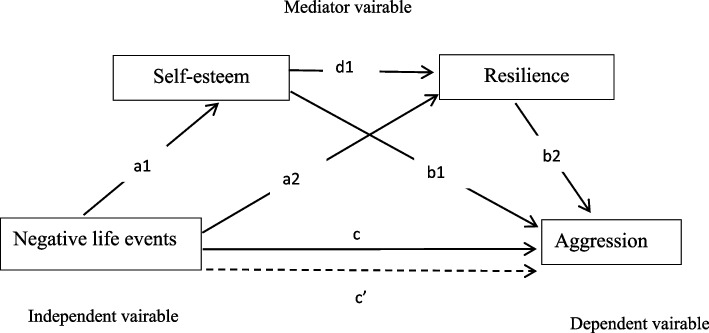
Hypothesized serial multiple mediation model. *Note:* a_1_: direct effect of negative life events on self-esteem, a2: direct effect of negative life events on resilience, b1: direct effect of self-esteem on aggression, b2: direct effect of resilience on aggression, d1: direct effect of self-esteem on resilience, c: total effect of negative life events on aggression, c’: direct effect of negative life events on aggression. **p < 0.001

Building and expanding on previous findings, we propose a model of the impact of negative life events on aggression that includes resilience and self-esteem as potential mediators based on Jiang Qianjin’s stress system model [33]. The stress system model suggests that there are many intermediary variables between stressors (life events) and stress reactions (psychological, behavioral and physiological outcome), including cognitive evaluation, social support, personality and other psychological factors [33]. Following this model, it is hypothesized that resilience and self-esteem (psychological factors) are mediators of the relationship between negative life events (stressors) and aggression (behavioral outcome) after considering context variables (e.g., demographics). A theoretical hypothesis model is shown in Fig. 1. We propose the following hypotheses: (1) negative life events are negatively related to self-esteem and resilience, while negative life events are positively related to aggression; (2) resilience and self-esteem are positively related to aggression; and (3) self-esteem mediates the relationship between negative life events and aggression via resilience.

## Methods

### Study population and procedure

A cross-sectional study with a stratified random sampling method was conducted among Chinese rural LBAs in Hunan Province from April 13 to April 20, 2020. The study was approved by the ethics committee of our university (Number: E201946). First, one city in each of the five administrative regions of Hunan Province was selected randomly. Next, we randomly chose one rural junior high school in each sampled city. Finally, we chose five rural junior high schools. We obtained permission from the principal and master teacher of each school before collecting the data. Students were gathered in several classrooms, and questionnaires were distributed with the help of the head teacher. Before filling out the questionnaires, all participants were informed about the purpose of the study, and an informed consent form was signed by the teacher who obtained parents’ authorization. The whole process was conducted completely voluntarily, anonymously, and confidentially. Furthermore, the participants could decline to participate in the study at any time without penalty. Students in selected classrooms completed the survey without the teacher present. The questionnaire took approximately 30–45 min to complete. Before the students completed the questionnaire, they were told to answer each question honestly and carefully and that the questionnaire results would only be used for scientific research. After all of the questionnaires were collected, we divided the students into LBA or non-LBA groups according to the answer to the question, “Did one or both of your parents out-migrate for work for more than 6 months?” We selected the LBA questionnaires [5]. The sample size was calculated according to 5-10 times the total items of the questionnaire. Considering a 20% loss of access rate and sampling error [34–36], the sample size was expanded to 1344.

#### Participants

The inclusion criteria for participants were as follows: (a) one or both of their parents were out-migrant workers for at least 6 months; (b) aged between 11 and 18 years; and (c) they volunteered for the study. Correspondingly, the exclusion criteria were as follows: (a) participants who were illiterate; (b) participants with severe mental disorders and inadequate communication ability assessed by psychiatrists; and (c) participants with hearing or speech dysfunctions.

Informed consent was obtained from eligible participants and their guardians. We initially recruited 1344 LBAs for the study. However, many items of the questionnaire were missing or empty; therefore, after excluding invalid questionnaires (one or more items of the main scales were empty), only 1294 students had completed all the questions and were included in the analysis.

#### Measures

##### Demographic characteristics questionnaire

Sociodemographic characteristics were measured by a self-designed questionnaire including LBAs’ age, gender, grade, yearly income, contact frequency with parents, parents’ education level, parents’ occupation, parents’ migrant status for work and having only one child. Details about sociodemographic information were collected by the following questions: As far as you know, what is the total annual income surplus of your family; What is the main source of family income; Are you the only child in your family; Did your father or mother out-migrate for work; Please choose your father’s/mother’s education level from the following options; Please choose your father’s/mother’s occupation from the following options; How often do you contact your parents by phone.

##### Adolescent Self-Rating Life Events Checklist (ASLEC)

The degree of negative event-induced stress was measured by the ASLEC, which consisted of 27 items in six factors, including interpersonal relationships, study pressure, punishment, sense of loss, healthy adaptation, and other factors. The Chinese scale was compiled by Liu Xianchen in 1997 [37] and used to assess the frequency and intensity of negative life events that may produce psychological effects on adolescents [38]. Each item was evaluated on a six-point Likert scale ranging from 0 (did not occur) to 5 (extremely severe). The total scores for the ASLEC were obtained with the total scores of the subscales. A higher score indicated greater stress related to negative life events. The Cronbach’s alpha coefficient of the ASLEC total scale in the original version was 0.85. In this study, it was 0.92.

##### Aggression Questionnaire (AQ)

The AQ was revised and completed by Arnold H. Buss in 2000 and translated into Chinese in 2008. The Chinese version of the AQ has shown good reliability and validity [39]. The scale includes 34 items that cover 5 components: physical aggression, verbal aggression, anger, hostility, and indirect aggression. Each item was evaluated on a five-point Likert scale ranging from 1 (extremely uncharacteristic of me) to 5 (extremely characteristic of me). Question 19 was scored ranging from 1 (extremely characteristic of me) to 5 (extremely uncharacteristic of me). The total scores for aggression were obtained with the total score of the subscales [40]. High scores represent higher levels of aggression behavior, with Cronbach’s alpha coefficients ranging from 0.61 to 0.81 in this study.

##### Resilience Scale for Chinese Adolescents (RSCA)

The RSCA was used to measure resilience. It was developed by HU in 2008, and its Cronbach’s alpha coefficients of the separate factor and total scale are all higher than 0.70 [41]. The scale includes 27 items that cover 5 components: goal focus, emotional control, positive cognition, family support and interpersonal assistance. Each item is scored with a 5-grade scoring method (1 = strongly disagree to 5 = strongly agree). The total scores are the sum of all the items, and higher scores indicate a higher level of resilience, with a Cronbach’s alpha coefficient of 0.84 in this study.

##### Rosenberg Self-esteem Scale (RSES)

Participants completed the widely used RSES [42], which measures global self-esteem. The Chinese version of the RSES was translated by Ji Yifu in 1993 and has shown good reliability and validity [43]. The RSES includes 10 items. It is a Likert scale with items answered on a four-point scale from strongly agree to strongly disagree. Some items are reverse scored; the higher the scores are for these items, the lower the respondent’s self-esteem. High scores represent higher levels of self-esteem, with Cronbach’s alpha coefficients ranging from 0.77 to 0.88 in this study.

### Statistical analysis

Data were entered into and analyzed using SPSS 26.0. First, we checked for missing values, outliers, and normality and excluded 50 missing data points before data analysis. Second, summative score values for each scale were calculated, and the total scores of ASLEC, AS, RSES and RSCA were approximately normally distributed. T tests and ANOVA were used to determine the associations between parent- and adolescent-related characteristics and aggression. The relationships between variables were examined via Pearson correlation analysis. Third, the PROCESS macro for SPSS was used to analyze the hypothesized mediation model, the approach based on ordinary least-squares regression and the bootstrap method [44]. Bootstrapping does not require the assumption of normality of the sampling distribution, and it has higher power while maintaining reasonable control over the Type I error rate [45]. Non-standardized beta coefficients are calculated to reduce Type 1 errors due to distribution. Pairwise contrasts are used to assess whether the three specific indirect effects are significantly different from one another comparisons if the 95% bias-corrected and accelerated confidence interval (CI) do not cross zero [45]. A previous study noted that Hayes recommended that 10,000 bootstrap samples be used for mediation analyses in the test from serial multiple mediation Model 6 [44]. We used 10,000 bootstrap resamples to calculate the 95% CI. If the interval did not include zero, the effect was statistically significant at *p* < 0.05.

## Results

### Characteristics of participants and differences in aggression, negative life events, self-esteem and resilience

As shown in Table 1, the participants’ ages ranged from 11 to 16 years (mean = 13.40, SD = 0.93). Of the total participants, 52.16% were male, 47.84% were female, the majority (84.39%) were not the only child in the family, and 59.12% of LBAs’ fathers out-migrated for work.

**Table 1:** Participants characteristics and differences in participants’ aggression, negative life events, resilience and self-esteem [M(SD)]

Variable	N (%)	Aggression	***t / F***	***P***	Negative life events	***t / F***	***P***	Resilience	***t / F***	***P***	Self-esteem	***t / F***	***P***
**Gender**													
Male	675 (52.16)	81.50 (24.51)	*t =* 1.789	0.074	46.17 (23.58)	*t =* −0.329	0.742	89.02 (13.91)	*t =* 0.241	0.810	27.70 (5.31)	*t =* − 0.192	0.847
Female	619 (47.84)	79.16 (22.60)			46.61 (24.58)			88.83 (14.50)			27.76 (5.04)		
**Grade**													
Seventh grade	437 (33.77)	78.13 (23.93)	*F =* 3.980	0.019*	46.73 (25.10)	*F =* 0.112	0.894	89.02 (14.88)	*F =* 0.105	0.986	27.39 (5.39)	*F =* 1.706	0.182
Eighth grade	458 (35.39)	80.48 (24.49)			45.98 (22.37)			88.91 (13.01)			27.86 (4.86)		
Ninth grade	399 (30.84)	82.73 (22.03)			46.47 (24.79)			88.86 (14.72)			28.01 (5.28)		
**Annual income surplus (in RMB)**													
<10,000	189 (14.61)	80.06 (24.08)	*F =* 2.837	0.037*	50.24 (25.36)	*F =* 1.908	0.126	87.97 (15.14)	*F =* 8.846	0.000**	27.69 (5.16)	*F =* 1.726	0.160
10,000-20,000	194 (14.99)	76.13 (21.86)			45.77 (25.26)			92.59 (15.93)			28.13 (6.05)		
>20,000	184 (14.22)	80.12 (24.79)			45.96 (21.89)			91.41 (13.84)			28.32 (5.06)		
Unclear	727 (56.18)	81.66 (23.55)			45.65 (23.86)			87.58 (13.27)			27.48 (4.95)		
**Source of income**													
Agriculture	111 (8.58)	78.42 (23.07)	*F =* 0.656	0.579	45.65 (24.93)	*F =* 3.566	0.014*	88.44 (13.37)	*F =* 2.133	0.094	28.80 (5.26)	*F =* 7.795	0.000**
Business	189 (14.61)	81.02 (27.01)			44.08 (24.98)			91.34 (15.69)			29.08 (5.35)		
Out-migrant for work	803 (62.05)	80.85 (22.88)			47.98 (24.06)			88.54 (13.90)			27.35 (5.03)		
Others	191 (14.76)	78.90 (23.43)			42.38 (22.02)			88.47 (14.13)			27.36 (5.28)		
**Only one child**													
Yes	202 (15.61)	79.13 (27.25)	*t* = −0.817	0.414	46.58 (25.79)	*t* = 0.130	0.897	89.84 (14.09)	*t* = 0.988	0.324	28.09 (5.09)	*t* = 1.077	0.282
No	1092 (84.39)	80.61 (22.89)			46.35 (23.73)			88.76 (14.20)			27.66 (5.19)		

*P < 0.05, **P < 0.001

Tables 1 and 2 show that LBAs of different grades (*F* = 3.980, *P* = 0.019), annual family income (*F* = 3.980, *P* = 0.019), father’s educational level (*F* = 3.712, *P =* 0.011*)*, mother’s occupation (*F* = 2.772, *P* = 0.026) and contact frequency with parents (*F* = 5.383, *P* < 0.001) had significant differences in aggression scores. The results showed that participants in higher grades, with higher annual family income and with less contact frequency with parents showed higher levels of aggression. Similarly, higher levels of aggression were found in LBAs with a higher level of father’s education, and lower contact frequency with parents. The higher the educational level of parents was the greater the contact frequency with LBAs, the higher the total scores were for resilience and self-esteem.

**Table 2:** Parents characteristics and differences in participants’ aggression, negative life events, resilience and self-esteem [M(SD)]

Variable	N (%)	Aggression	***t / F***	***P***	Negative life events	*t / F*	*P*	Resilience	t / F	*P*	Self-esteem	***t / F***	*P*
**Father goes out-migrant for work**													
Yes	765 (59.12)	80.99 (23.20)	*t* = 1.109	0.268	49.29 (23.95)	*t* = 5.283	0.000**	88.32 (14.42)	*t* = −1.877	0.061	27.36 (5.19)	*t* = −3.063	0.002**
No	529 (40.88)	79.50 (24.20)			42.18 (23.60)			89.82 (13.81)			28.26 (5.12)		
**Mother goes out-migrant for work**													
Yes	557 (43.04)	81.17 (23.11)	*t* = −1.051	0.293	48.62 (23.31)	*t* = 2.916	0.004**	87.87 (13.72)	*t* = −2.338	0 .020*	27.17 (5.04)	*t* = −3.363	0.001**
No	737 (56.96)	79.78 (23.99)			44.69 (24.48)			89.73 (14.48)			28.15 (5.24)		
**Father’s educational level**													
Below primary school	167 (12.91)	79.64 (23.74)	*F =* 3.712	0.011*	47.38 (23.27)	*F =* 8.059	0.000**	85.31 (14.27)	*F =* 0.676	0.000**	27.04 (4.64)	*F =* 2.896	0.034*
Junior high school	774 (59.81)	81.84 (23.27)			48.20 (24.15)			88.76 (13.72)			27.60 (5.16)		
High school	297 (22.95)	76.62 (24.09)			40.46 (23.00)			91.31 (15.02)			28.40 (5.24)		
University or above	56 (4.33)	82.39 (23.68)			49.70 (26.03)			89.46 (13.67)			28.02 (6.29)		
**Mother’s educational level**													
Below primary school	209 (16.15)	83.15 (22.93)	*F =* 1.154	0.326	49.22 (23.52)	*F =* 1.251	0.290	85.24 (14.62)	*F =* 7.051	0.000**	26.49 (4.80)	*F =* 4.964	0.002**
Junior high school	765 (59.12)	79.78 (23.85)			45.96 (23.25)			89.09 (13.67)			27.90 (5.09)		
High school	250 (19.32)	79.95 (23.39)			45.20 (26.63)			90.86 (14.90)			28.18 (5.43)		
University or above	70 (5.41)	80.17 (23.76)			46.74 (24.47)			91.34 (13.99)			27.99 (5.86)		
**Father’s occupation**													
Worker	596 (46.06)	79.68 (22.18)	*F =* 0.340	0.851	46.94 (23.62)	*F =* 0.603	0.661	88.30 (13.75)	*F =* 2.021	0.089	27.41 (4.97)	*F =* 2.126	0.075
Farmer	189 (14.61)	80.26 (23.93)			46.43 (23.97)			88.73 (13.97)			27.77 (4.94)		
Public servant	42 (3.24)	80.52 (23.08)			45.67 (31.05)			91.81 (16.22)			27.48 (6.94)		
Businessman	198 (15.30)	80.91 (27.84)			44.03 (25.15)			91.13 (16.39)			28.64 (5.67)		
Others	269 (20.79)	81.61 (23.31)			46.96 (23.08)			88.41 (13.05)			27.76 (5.07)		
**Mother’s occupation**													
Worker	373 (28.83)	78.07 (23.41)	*F =* 2.772	0.026*	45.23 (24.09)	*F =* 2.085	0.081	88.25 (13.63)	*F =* 2.527	0.039*	27.39 (5.20)	*F =* 1.898	0.108
Farmer	308 (23.80)	80.25 (23.12)			46.09 (24.05)			89.79 (14.66)			28.38 (4.91)		
Public servant	79 (6.10)	76.78 (22.43)			44.85 (25.91)			92.67 (15.90)			27.54 (6.41)		
Businessman	202 (15.61)	81.36 (24.77)			44.39 (23.71)			89.39 (15.04)			27.88 (5.17)		
Others	332 (25.66)	83.36 (23.62)			49.53 (23.62)			87.73 (13.23)			27.47 (5.04)		
**Contact frequency**													
Every 3 days	378 (29.21)	79.26 (24.90)	*F =* 5.383	0.000**	42.05 (23.72)	*F =* 16.556	0.000**	89.49 (14.53)	*F =* 15.031	0.000**	28.36 (5.25)	*F =* 12.495	0.000**
Every 1 week	281 (21.72)	76.77 (24.28)			41.41 (23.60)			92.51 (14.36)			28.80 (4.97)		
Every 2 weeks	370 (28.59)	80.52 (22.80)			48.35 (23.36)			89.52 (14.05)			27.50 (5.25)		
Every 3 weeks	68 (5.26)	87.90 (23.76)			51.93 (23.17)			83.97 (10.99)			26.37 (4.32)		
Every 1 month or above	197 (15.22)	84.81 (20.32)			56.20 (23.28)			83.38 (12.43)			25.88 (4.85)		

*P < 0.05, **P < 0.001

### Correlation between aggression, negative life events, self-esteem and resilience

The basic descriptive data for aggression, negative life events, self-esteem and resilience are shown in Table 3. The mean total aggression score was 80.38 ± 23.62 (range = 34-170), negative life event score was 46.38 ± 24.055 (range = 0-135), self-esteem score was 27.37 ± 5.31 (range = 10-60), and resilience score was 88.93 ± 14.19 (range = 27-135). Pearson’s correlations showed that negative life events were significantly negatively correlated with self-esteem (*r* = − 0.338, *p* < 0.001) and resilience (*r* = − 0.359, *p* < 0.001). Additionally, negative life events were significantly positively correlated with aggression (*r* = 0.441, *p* < 0.001).

**Table 3:** Descriptive statistics and correlation of aggression, negative life events, resilience and self-esteem (*N* = 1294)

Item	Range	M ± SD	95%CI	1	2	3	4
**1 Aggression**	34–170	80.38 ± 23.62	79.09–81.67	1			
**2 Negative life events**	0–135	46.38 ± 24.06	45.07–47.69	0.441**	1		
**3 Resilience**	27–135	88.93 ± 14.19	88.16–89.70	−0.383**	−0.359**	1	
**4 Self-esteem**	10-60	27.73 ± 5.18	27.45–28.01	−0.323**	−0.338**	0.581**	1

**P < 0.001

### Mediating role of self-esteem and resilience on negative life events and aggression

**Fig. 2:**
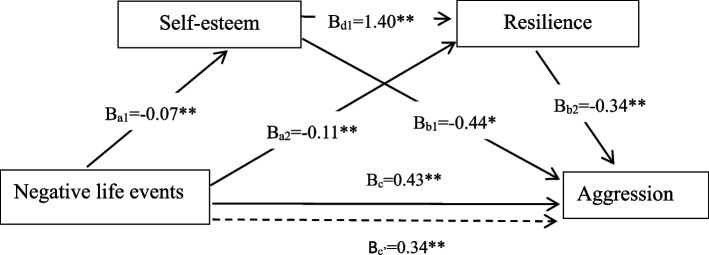
Serial multiple mediation model with self-esteem and resilience as mediators in the relationship between negative life events and aggression

Table 4 shows that all individual paths between the key variables in the model were significant after accounting for covariates. Higher levels of negative life events were associated with lower self-esteem (*β* = − 0.07, 95% CI: − 0.080 ~ − 0.055) and resilience (*β* = − 0. 11, 95% CI: − 0.137 ~ − 0.076). Both higher self-esteem and resilience were also associated with lower aggression (*β* = − 0.44, 95% CI: −.745 ~ − 0.132; *β* = − 0.34, 95% CI: − 0.452 ~ − 0.229, respectively). Additionally, the total effect (Bc) and the total direct (Bc’) effect of negative life events on aggression were found to be significant (B_c_ = 0.43, SE = 0.03, t = 16.88, *p* < 0.001; B_c’_ = 0.34, SE = 0.03, t = 10.69, *p* < 0.001), as shown in Fig. 2.

**Table 4:** Results of the regression analyses testing the serial multiple mediation effect of resilience and self-esteem in the relationship negative life events and aggression (*N* = 1294)

Predictors	Direct effect (SE)			Total effect (SE)
	Self-esteem	Resilience	Aggression	Aggression
Constant	31.08 (0.78)**	56.86 (3.04) **	95.97 (6.38) **	48.18 (3.24) **
Negative life events	−0.07 (0.01)**	− 0. 11 (0.02) **	0.34 (0.03) **	0.43 (0.03) **
Self-esteem		1.40 (0.07) **	−0.44 (0.16) *	
Resilience			−0.34 (0.06) **	
Grade	0.35 (0.17)*	−0.55 (0.40)	2.69 (0.69) **	2.55 (0.71) **
Annual income surplus (in RMB)	−0.20 (0.12)	−0.73 (0.28) *	1.25 (0.48) *	1.68 (0.50) **
Father’s educational level	0.34 (0.20)	1.05 (0.45) *	0.45 (0.82)	−0.22 (0.84)
Mother’s occupation	0.01 (0.08)	−0.06 (0.19)	0.77 (0.36)*	0.78 (0.37)*
Contact frequency	−0.41 (0.10)**	−0.25 (0.24)	− 0.46 (0.43)	0.01 (0.44)
*R*^2^	0.13**	0.38**	0.27**	0.21**

Unstandardized regression coefficients (beta) with standard error (SE) in parentheses are presented

**P < 0.001

According to the above findings, three paths were used to determine the effect of negative life events on aggression (see Table 5). Table 5 shows that there was a significant sequential indirect effect of negative life events on aggression through self-esteem and resilience [path a1d1b2: Effect = 0.0322, SE =0.0066; 95% CI (0.0207, 0.0473)]. Additionally significant were the simple mediation paths from negative life events to aggression via self-esteem [path a1b1: Effect = 0.0296, SE =0.0110; 95% CI (0.0094, 0.0526)] and from negative life events to aggression through resilience [path a2b2: Effect = 0.0361, SE = 0.0087; 95% CI (0.0213, 0.0562)]. The separate mediating effects and the serial-multiple mediation between the three models were not at a zero-point estimate interval within the 95% CI, and self-esteem and resilience had significant mediating effects on aggression. The direct effect of negative life events on aggression was 0.3364, accounting for just 77.44% of the total effect (0.4344). Second, the total indirect effect of negative life events on aggression through self-esteem and resilience was 0.0980, which accounted for 22.56% of the total effect (0.4344).

**Table 5:** Bootstrapped point estimates with standard errors and 95% confidence intervals for all indirect effects between negative life events and aggression (*N* = 1294)

				Bootstrapping 95% CI	
	(Path)	Effect	SE	Lower	Upper
**Direct effects**	(c’)	0.3364	0.0315	0.2747	0.3982
**Indirect effects**					
Via self-esteem	(a1b1)	0.0296	0.0110	0.0094	0.0526
Via resilience	(a2b2)	0.0361	0.0087	0.0213	0.0562
Via self-esteem and resilience	(a1d1b2)	0.0322	0.0066	0.0207	0.0473
**Total indirect effect**		0.0980	0.0150	0.0699	0.1296
**Total effect**		0.4344	0.0257	0.3839	0.4849

If the CI does not include zero, the effect is statistically significant at *p* < 0.05

*Abbreviations*: *CI* Confidence interval, *SE* Standard error

## Discussion

### Descriptive analysis and social characteristics of participants for aggression, negative life events, self-esteem and resilience

The present study found a relatively high level of aggression, with a result similar to Huang’s study [46]. Additionally, we found a significant difference in aggression with regard to grade, annual income of families, parents’ educational level, mother’s occupation and contact frequency. LBAs who were in higher grades had more aggressive behavior. A possible reason for the significant difference in grade is that children who are in lower grades are not familiar with the environment, and there is no great friction between them. Children who are in higher grades have become used to the school environment and activities, and some of them have formed small groups, which are prone to aggressive behavior when they disagree with something [5]. With regard to the socioeconomic status of families, consistent with Huang’s study [46], adolescents from poor families were not more likely to have higher aggression rates than those from wealthy families in Chinese rural areas. A possible reason for this discrepancy is the characteristics of the monitoring type in China [46]. Previous research results show that the proportion of grandparent care of left-behind children is significantly higher than that of non-left-behind children (30.90% vs. 6.23%) [47]. Some left-behind children are supervised by grandparents, and the grandparents spoil the children and give them more materialistic things, so the children do not receive enough spiritual and moral guidance. The results showed that LBAs who had less frequent contact with parents showed higher levels of aggression. This was also the case for LBAs who had fathers with a higher education level, lower contact frequency with parents, and higher scores for negative life events. The higher the educational level of parents was, the greater the contact frequency with LBAs and the higher the total scores were for resilience and self-esteem. Many parents of LBAs were absent for a long time, and the reduction in contact frequency with their parents made them lack parental care [48]. These children could not obtain guidance in the face of conflict and setbacks, could not resolve negative emotions such as anger, hostility and worry, manifested these concerns in disputes, complaints, blame and ridicule, and were more prone to be aggressive [49].

### Correlation between aggression, negative life events, self-esteem and resilience

Our study showed that negative life events of LBAs were significantly positively related to aggression, consistent with previous studies [23, 46]. Anderson’s “general aggression model” notes that stressful life events are primary circumstantial factors that are significantly associated with aggression [21]. The stimulation of some negative life events, such as study pressure, punishment, and interpersonal relationships, may aggravate the level of emotional arousal without timely help and support from parents, leading adolescents to engage in aggression [5]. At the same time, previous studies on aggression have noted that negative life events usually occur in individuals with poor academic performance and interpersonal communication, and these people are more likely to have a high level of reactive aggression [50].

### Mediating role of self-esteem and resilience on negative life events and aggression

The study sought to explain the mediating role of self-esteem and resilience between negative life events and aggression based on the stress system model. Our study showed that negative life events not only have direct effects on aggression but also have indirect effects on aggression via self-esteem and resilience. These new findings are consistent with our hypotheses and similar to those of previous studies [51, 52]. According to the frustration aggression hypothesis model, when an individual is frustrated in achieving a desired goal, aggressive behavior will be aroused in a specific situation [53, 54]. This is especially true for unexpected life events, and negative life events are considered an important external environmental factor that stimulates the formation of reactive aggression [55]. Additionally, our study showed that negative life events affect aggression via self-esteem and resilience. This is consistent with Wu’s study [56]. Low self-esteem is considered an important predictor of aggressive behavior, which is closely related to the formation of aggression [57]. Furthermore, in aggression-related research, self-esteem is usually regarded as an individual’s motivation to display aggressive behavior, and lower self-esteem can cause great anxiety in people with aggressive tendencies, even evoking a sense of frustration and shame [58, 59]. So that individuals with low self-esteem use aggressive responses to compensate for their negative emotions [38]. Furthermore, the stimulation intensity of negative life events for individuals is related not only to the negative events themselves but also to the differences in emotional stability among different individuals. Resilience plays a very important role in this process. The results showed that people with a higher level of resilience tend to adopt less aggression due to negative life events. This finding is similar to previous research [10, 60, 61]. Kumpfer’s resilience model suggests that resilience is one of the most significant factors for people in dealing with stress events [25]. People with high resilience may have more psychological advantages (optimism, self-esteem and self-confidence) and accessible resources (social support) [62]. These factors can help individuals achieve good adaptation in adverse environments [63].

Our study had several limitations. First, students were recruited from only one province of China. Thus, the findings of this study may not be generalizable to all LBAs. Further research should be conducted using multiarea research. Second, our study was a cross-sectional study; future longitudinal studies should be conducted to better elucidate the dynamic connection between negative life events, aggression, resilience and self-esteem. Finally, positive psychological resources were measured only by resilience and self-esteem in our study. However, positive psychological resources also include other aspects, such as well-being measures [64]. Therefore, more positive psychological measures should be included in future studies to enrich our results. Nevertheless, unlike a previous study [10], our research clarified the specific negative social adjustment situation (aggression) of LBAs and explored the mediating variables that can alleviate aggressive behavior due to negative life events from positive psychology, which provides a new perspective for the mental health management intervention of LBAs. It is inevitable for LBAs to encounter negative life events during their development, and the absence of their parents aggravates this phenomenon. This phenomenon is difficult to change in the short term in rural areas of China. Some studies have confirmed that individual resilience can be improved through scientific intervention methods [65, 66], such as cognitive behavioral therapy [66], painting therapy [67] and peer education [68]. In addition, families, schools and society should work together to create a harmonious environment for the healthy development of LBAs.

The ecological systems theory propounded in 1979 by Urie Bronfenbrenner suggests that human growth and development are influenced by various systems, including the microsystem (parent and school), mesosystem (family and community) and macrosystem (social environment, ideology and culture) [69]. Parents should attach importance to learning scientific approaches to education, such as by actively communicating with teachers or older people about children’s life and studies. School is an important part of the supportive psychological care system for LBAs. Online psychological health education courses can be established for family members. In addition, schools can set up psychological counseling rooms. Public welfare groups or relevant organizations can actively mobilize volunteers to participate in caring services and assistance activities for LBAs to help solve problems in studies and life.

## Conclusions

In this study, we found that resilience and self-esteem played a mediating role between negative life events and aggression among Chinese rural LBAs. Furthermore, negative life events negatively affected self-esteem and resilience and positively affected aggression in LBAs, whereas self-esteem and resilience played a protective role between negative life events and aggression. The research demonstrated preliminary relationship mechanisms for negative life events, self-esteem, resilience and aggression in LBAs. The results provide a reference for policy-makers, educators, and intervention program designers to reduce the occurrence of aggression by enhancing LBAs’ self-esteem and resilience.

## Data Availability

The data sets used and /or analyzed during the current study are available from the corresponding author on reasonable request. The data are not publicly available due to privacy or ethical restrictions.

## Abbreviations

LBAs: Left-behind adolescents
ASLEC: Adolescent Self-Rating Life Events Checklist
AS: Aggression Scales
RSCA: Resilience Scale for Chinese Adolescent
RSES: Rosenberg Self- esteem Scale
SE: Standard errors
CI: Confidence interval
